# Assessment of biochemical markers in the early post-burn period for predicting acute kidney injury and mortality in patients with major burn injury: comparison of serum creatinine, serum cystatin-C, plasma and urine neutrophil gelatinase-associated lipocalin

**DOI:** 10.1186/cc13989

**Published:** 2014-07-14

**Authors:** Hyeong Tae Yang, Haejun Yim, Yong Suk Cho, Dohern Kym, Jun Hur, Jong Hyun Kim, Wook Chun, Hyun Soo Kim

**Affiliations:** 1 Department of Surgery, Burn center, Hallym University Hangang Sacred Heart Hospital, Hallym University College of Medicine, 12, Beodeunaru-ro 7-gil, Youngdeungpo-gu, Seoul 150-719, Korea; 2 Department of Laboratory Medicine, Hallym University Dongtan Sacred Heart Hospital, Hallym University College of Medicine, 7, Keunjaebong-gil, Hwaseong-si, Gyeonggi-do 445-170, Korea; 3 Department of Surgery, Kangwon National University College of Medicine, 1 Kangwondaehak-gil, Chuncheon-si, Gangwon-do 200-701, Korea

## Abstract

**Introduction:**

The reported mortality rates range from 28% to 100% in burn patients who develop acute kidney injury (AKI) and from 50% to 100% among such patients treated with renal replacement therapy. Recently, the serum cystatin C and plasma and urine neutrophil gelatinase-associated lipocalin (NGAL) levels have been introduced as early biomarkers for AKI; the levels of these biomarkers are known to increase 24 to 48 hours before the serum creatinine levels increase. In this study, we aimed to estimate the diagnostic utility of the cystatin C and plasma and urine NGAL levels in the early post-burn period as biomarkers for predicting AKI and mortality in patients with major burn injuries.

**Methods:**

From May 2011 to July 2012, 90 consecutive patients with a burn wound area comprising ≥ 20% of the total body surface area (TBSA) were enrolled in this study. Whole blood and urine samples were obtained for measuring the serum creatinine, serum cystatin C, and urine and plasma NGAL levels at 0, 3, 6, 12, 24, and 48 hours after admission. Receiver operating characteristic curve, area under the curve, and multivariate logistic regression analyses were performed to assess the predictive values of these biomarkers for AKI and mortality.

**Results:**

In the multivariate logistic regression analysis, all variables, including age, percentage TBSA burned, sex, inhalation injury, and serum creatinine levels, serum cystatin C levels, and plasma and urine NGAL levels were independently associated with AKI development. Moreover, age, sex, percentage TBSA burned, and plasma and urine NGAL levels were independently associated with mortality. However, inhalation injury and the serum creatinine and cystatin C levels were not independently associated with mortality.

**Conclusions:**

Massively burned patients who maintained high plasma and urine NGAL levels until 12 hours after admission were at the risk of developing early AKI and early mortality with burn shock. However, the plasma and urine NGAL levels in the early post-burn period failed to predict late AKI and non-burn shock mortality in this study. Nevertheless, the plasma and urine NGAL levels were independently associated with AKI development and mortality within 48 hours after admission.

## Introduction

The reported incidence rates of acute kidney injury (AKI) among burn patients range from <1 to 36%, depending on the population studied and the classification criteria used [1]. The reported mortality rates among burn patients who develop AKI range from 73 to 100% [2]. During the early phase of a major burn injury, the most common cause of AKI is ischemic organ damage from effective hypovolemia caused by a massive systemic inflammatory response. During the late phase of a major burn injury, sepsis and nephrotoxic agents are the most common causes of AKI.

Laboratory research has revealed that early intervention may be essential for preventing the pathophysiologic events that lead to AKI. However, serum creatinine, which is one of the main AKI biomarkers used in clinical settings, is a late marker for reduced glomerular filtration rate, which limits its use in early AKI detection and clinical therapeutic studies [3]. Recently, the levels of serum cystatin C and plasma and urine neutrophil gelatinase-associated lipocalin (NGAL) have been suggested as early biomarkers for AKI as their levels were found to increase 24 to 48 h prior to an increase in the serum creatinine level [4-12].

Few studies have elucidated the associations of the serum cystatin C, plasma NGAL, and urine NGAL levels with AKI in patients with major burn injuries. This study had the following aims: (1) to investigate the levels of serum creatinine, cystatin C, and plasma and urine NGAL over time during the early post-burn period; (2) to estimate the diagnostic utility of the cystatin C and plasma and urine NGAL levels for predicting AKI and mortality in patients with major burn injuries; and (3) to determine the relationships between the levels of these biomarkers and the burn sizes and inhalation injuries, which are known prognostic factors for burn injuries.

## Materials and methods

### Patient selection and subgroups

From May 2011 to July 2012, 90 consecutive patients were enrolled in this prospective cohort study. The study protocol was approved by the Institutional Review Boards of Hangang Sacred Heart Hospital (IRB number 2011–143), and informed consent was obtained from all subjects. None of the patients were lost to follow up. The inclusion criteria were as follows: patients aged ≥18 years with % total body surface area (TBSA) burned ≥20%. Because we intended to investigate changes in biomarker levels during the early post-burn period, we limited the inclusion criteria to those patients who were admitted to our burn intensive care unit within 6 h of injury. Patients with known cardiac disease (for example, prior history of heart failure, arrhythmia, or coronary heart disease), prior kidney transplant, end-stage kidney disease, or chronic liver disease (for example, prior history of liver cirrhosis or chronic hepatitis) were excluded. Multiple data, including sex, age, body weight, presence of co-morbidities, % TBSA burned, % TBSA with third-degree burn wounds, cause of burn injury, and presence of inhalation injury, were collected for each patient.

AKI diagnoses were made according to the risk, injury, failure, sustained loss, and end-stage kidney disease (RIFLE) criteria [13]. These criteria include an increase in the serum creatinine level ≥ 50% over the baseline or reduction in the urine output to < 0.5 mL/kg/h for a period > 6 h. The baseline renal function was defined as the lowest known serum creatinine value during the preceding 3 months; however, most of the patients had not undergone a previous laboratory evaluation. For patients without known prior serum creatinine levels, we used the lowest serum creatinine level measured during the admission period only when the level was within a normal range. However, the lowest serum creatinine levels were abnormal in six patients. In these cases, the baseline serum creatinine levels were estimated using the modified diet in renal disease equation for the assessment of kidney function while assuming a glomerular filtration rate of 75 mL/minute/1.73 m^2^[13]. Considering the patients’ ages, it is possible that we underestimated the AKI. However, all six patients were included in the AKI group with a RIFLE stage R. Therefore, AKI was not underestimated because of the baseline serum creatinine levels.

As described in the Introduction, the primary cause of AKI differs according to the time of AKI development. Thus, we classified the patients into the following three groups according to the time of AKI development: the no-AKI group; the early-AKI group, wherein AKI developed within 5 days of burn injury; and the late-AKI group, wherein AKI developed after 5 days post burn injury. The mean time from burn injury to early AKI development was 1.2 days. No patients developed AKI on day 4; however, one patient developed AKI on day 5 because of rhabdomyolysis caused by an electrical injury. Therefore, this patient was included in the early AKI patient group. Thus, early AKI was considered as AKI that developed up to 5 days after burn injury.

Additionally, we classified the patients as survivors or non-survivors according to mortality. The non-survivors with burn shock had experienced the most severe burn injuries, and their length of hospital stay was very short. Therefore, we decided to divide the patient groups into the following three groups based on the time of death: the early-death group, the late-death group, and the survival group. The early-death group included non-survivors who developed burn shock despite fluid resuscitation and died within 3 days of burn injury.

To determine whether the biomarker levels were affected by the burn size or presence of inhalation injury, we also classified the patients into two groups: smaller burn-size group (% TBSA burned, 20 to 49%) and larger burn-size group (% TBSA burned, 50 to 100%).

### Specimen collection and measurement of biomarkers

Blood samples were collected via a central venous catheter and urine samples were collected via a Foley catheter, at 0, 3, 6, 12, 24, and 48 h after admission to the burn intensive care unit. The blood NGAL levels were measured with the Triage NGAL reagent and Triage Meter (Alere Healthcare, San Diego, CA, USA). The serum cystatin C levels were measured according to a turbidimetric immunoassay method with the HiSense cystatin C kit (HBi, Anyang, Korea) and Hitachi 7600 analyzer (Hitachi, Tokyo, Japan). The serum and urine creatinine levels were measured according to an enzymatic method with the Cica Creatinine reagent (KANTO Chemical, Tokyo, Japan) and Hitachi 7600 analyzer (Hitachi, Tokyo, Japan).

For the urine NGAL analysis, urine specimens were transferred to centrifuge tubes and centrifuged at a relative centrifugal force ≥ 400 for a minimum of 5 minutes; the supernatants were stored at -70°C prior to batch analysis. After thawing, the specimens were mixed and centrifuged at 2,500 to 3,000 × g for 10 minutes prior to use, to remove any particulate matter and ensure consistency in the results. The urine NGAL levels were measured in a chemiluminescence immunoassay with an Architect i2000SR analyzer (Abbott Diagnostics, Abbott Park, IL, USA) and a dedicated urine NGAL reagent (Abbott Diagnostics). All measurements were performed according to the manufacturers’ instructions.

### Data analysis

**Figure 1:**
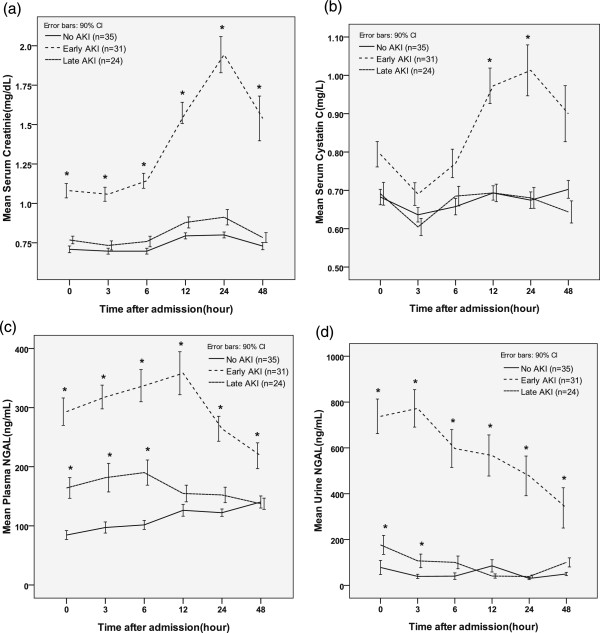
**Comparison of serum creatinine (a), cystatin C (b), plasma neutrophil gelatinase-associated lipocalin (NGAL) (c), and urine NGAL (d) levels at different time points with respect to acute kidney injury (AKI) development.** Early AKI, development of AKI within 5 days of injury; Late AKI: development of AKI beyond 5 days after injury. *Significant difference (*P* <0.05) relative to the no AKI group.

**Figure 2:**
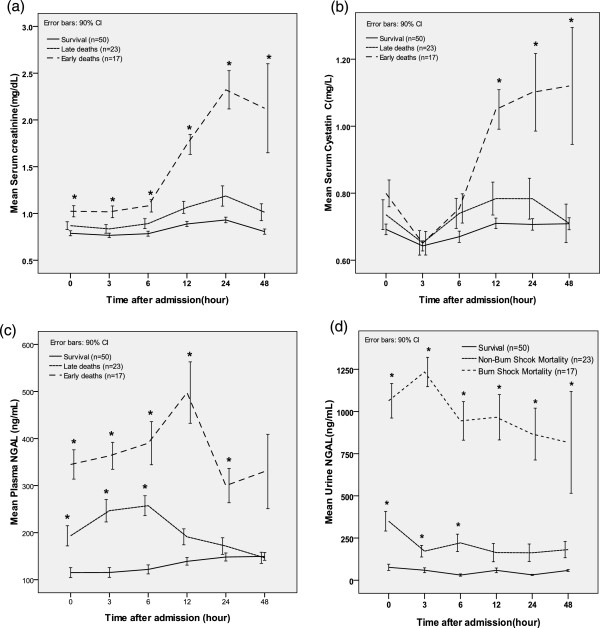
**Comparison of serum creatinine (a), cystatin C (b), plasma neutrophil gelatinase-associated lipocalin (NGAL) (c), and urine NGAL (d) levels at different time points with respect to mortality.** Early deaths: non-survivors who developed burn shock despite fluid resuscitation, and died within 3 days of burn injury. Late deaths: mortality due to causes other than burn shock beyond 3 days after burn injury. *Significant difference (*P* <0.05) relative to the survival group.

**Figure 3:**
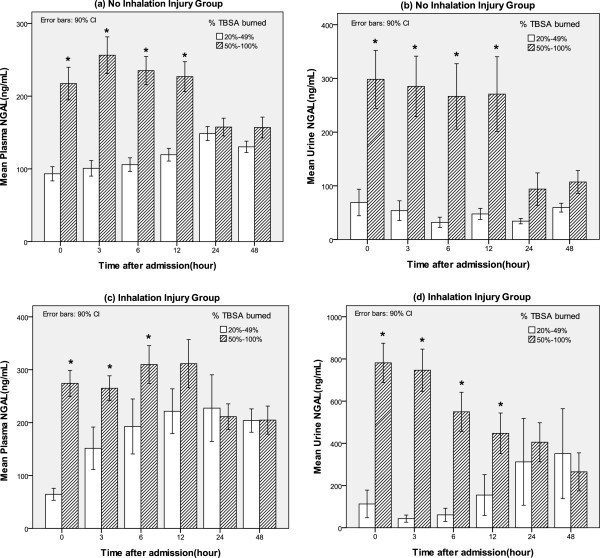
**Comparison of the plasma and urine neutrophil gelatinase-associated lipocalin (NGAL) levels according to burn size and the presence of inhalation injuries. (a, b)** No inhalation injury group. **(c, d)** Inhalation injury group. *Significant difference (*P* < 0.05) between groups. TBSA, total body surface area.

**Figure 4:**
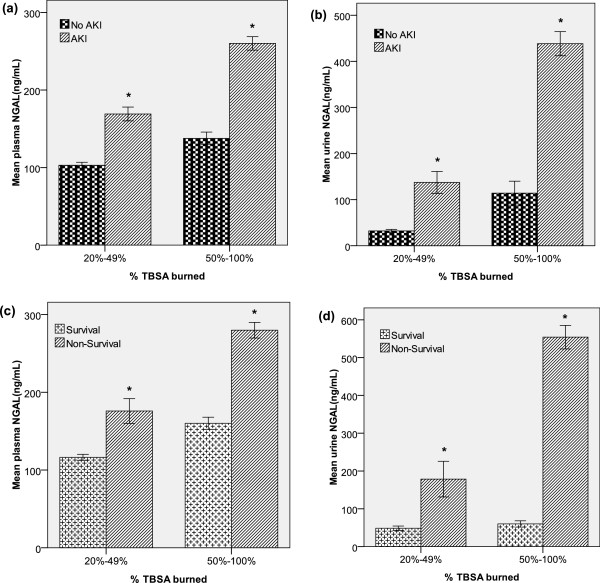
**Comparison of the plasma and urine neutrophil gelatinase-associated lipocalin (NGAL) levels according to acute kidney injury and mortality at all time points and according to the subgroup for percentage total body surface area (TBSA) burned. (a, b)** Acute kidney injury (AKI). **(c, d)** Mortality. *Significant difference (*P* < 0.05) between groups.

Data were expressed as mean ± SD or number (%) for each subgroup of AKI development and mortality (Tables 1 and 2). Statistical differences among subgroups were analyzed using the Mann–Whitney *U*-test or Kruskal-Wallis test. We investigated and compared changes in serum creatinine, cystatin C, and plasma and urine NGAL levels during the 48 h after admission in the groups classified according to the time of AKI development and mortality (Figures 1 and 2). Thereafter, comparisons of the mean serum creatinine, cystatin C, plasma NGAL, and urine NGAL levels at each point between patients with and without AKI and between survivors and non-survivors were performed and statistically analyzed. We also compared the changes in the levels of these biomarkers between the groups classified according to the burn size and inhalation injury, which are known prognostic factors for burn injury (Figures 3 and 4). The diagnostic abilities of the plasma and urine NGAL levels for predicting AKI and mortality were assessed by calculating the areas under the receiver operating characteristic curves (AUC-ROC) and the cut-off value was defined by Youden’s index (Tables 3 and 4). Multivariate analysis was performed to identify variables independently associated with AKI development and mortality (Table 5). SPSS 17.0 software (SPSS, Inc, Chicago, IL, USA) was used for statistical analysis and a *P-*value < 0.05 was considered statistically significant.

**Table 1:** Patient characteristics according to acute kidney injury (AKI) development

	**Non-AKI (n = 35)**	**AKI (n = 55)**		***P*-value**
		**Early AKI (n = 31)**	**Late AKI (n = 24)**	
Age, mean ± SD	45.4 ± 14.8	51.3 ± 15.5	52.5 ± 12.4	0.129
Sex, male:female, n (%)	29:6 (82:18)	28:3 (90:10)	20:4 (83:17)	0.641
Mode, FB:SB:EB:CB:SKB, n (%)	27:1:4:2:1 (77:3:12:5:3)	28:1:2:0:0 (91:3:6:0:0)	20:1:1:1:1 (84:4:4:4:4)	
TBSA burned, %, mean ± SD	38.4 ± 14.1	70.7 ± 22.0	61.5 ± 19.2	<0.001
Third-degree burn wound, %, mean ± SD	24.6 ± 10.2	62.5 ± 28.6	48.5 ± 21.3	<0.001
Inhalation injury, n (%)	4 (11.4)	17 (54.8)	8 (33.3)	0.01
Mechanical ventilation, n (%)	5 (14.3)	28 (90.3)	19 (79.2)	<0.001
Rhabdomyolysis, n (%)	7 (20.0)	24 (77.4)	4 (16.7)	<0.001
Early vasopressor, n (%)	1 (2.8)	21 (67.7)	3 (12.5)	<0.001
Post-burn day of AKI, days, mean ± SD		1.2 ± 0.6	18.9 ± 12.1	<0.001
CRRT, n (%)		7 (22.6)	15 (62.5)	<0.001
Duration of CRRT, days, mean ± SD		1.9 ± 0.7	10.9 ± 2.8	<0.001
Sepsis, n (%)	3 (8.6)	7 (22.6)	20 (83.8)	<0.001
LOS, day, mean ± SD	55.7 ± 23.1	26.6 ± 43.6	42.2 ± 35.2	0.003
Mortality, n (%)	2 (5.7)	24 (77.4)	14 (58.3)	<0.001
Maximal RIFLE score, n (R:I:F)		0:10:21	1:5:18	

Early AKI, AKI that developed within 5 days of injury; Late AKI, AKI that developed after 5 days post injury; FB, flame burn; SB, scalding burn; EB, electrical burn; CB, chemical burn; SKB, spark burn; TBSA, total body surface area; Early vasopressor, vasopressor infusion due to shock within 1 day of admission; CRRT, continuous renal replacement therapy; LOS, length of hospital stay; RIFLE, risk, injury, failure, sustained loss, and end-stage kidney disease; n, number of patients.

**Table 2:** Patient characteristics according to mortality

	**Survivors (n = 50)**	**Non-survivors (n = 40)**			**Total patients (n = 90)**
		**Early deaths (n = 17)**	**Late deaths (n = 23)**	**Total deaths (n = 40)**	
Age, mean ± SD	45.4 ± 14.2	54.5 ± 13.3	54.1 ± 14.5	54.3 ± 13.8	49.3 ± 13.5
Sex, male:female (%)	43:7 (86:14)	15:2 (88:12)	19:4 (83:17)	34:6 (85:15)	77:13 (86:14)
Mode, FB:SB:EB:CB:SKB (%)	40:1:5:3:1 (80:2:10:6:2)	15:1:1:0:0 (88:6:6:0:0)	20:1:1:0:1 (87:4:4:0:4)	35:2:2:0:1 (88:5:5:0:3)	75:3:7:3:2 (83:3:8:3:2)
TBSA burned, mean % ± SD	41.9 ± 15.2	82.9 ± 17.4	65.7 ± 18.4	73.0 ± 19.7	55.7 ± 23.2
Third-degree burn wound, mean % ± SD	28.0 ± 13.4	77.0 ± 24.2	54.4 ± 22.3	64.0 ± 25.5	44.0 ± 26.5
Inhalation injury, n (%)	11 (22)	13 (76.4)	5 (21.7)	18 (45)	29 (32.2)
Mechanical ventilation, n (%)	12 (24)	17 (100)	23 (100)	40 (100)	52 (57.8)
Rhabdomyolysis, n (%)	10 (20)	15 (88.2)	10 (43.4)	25 (62.5)	35 (38.9)
AKI, n (%)	17 (34)	17 (100)	21 (91)	38 (95)	55 (61.1)
Early AKI, n (%) (%)	7 (14)	17 (100)	6 (26)	23 (57.5)	31 (33.3)
Post-burn day of AKI, days, mean ± SD	14.0 ± 15.9	1.0 ± 0.0	11.2 ± 9.8	6.6 ± 8.9	9.1 ± 12.1
Cause of AKI, BS:RD:SS:MD, n	4:3:0:10	16:1:0:0	3:3:14:1	19:4:14:1	23:7:14:11
CRRT, n (%)	5 (10)	0 (0)	17 (73.9)	17 (42.5)	22 (24.4)
RIFLE (R:I:F) starting CRRT, n	0:0:5		2:8:7	2:8:7	2:8:12
Post-burn day of CRRT, days, mean ± SD	10.8 ± 7.6		14.9 ± 9.9	14.9 ± 9.9	14.0 ± 9.4
Duration of CRRT, days, mean ± SD	9.2 ± 4.4		7.0 ± 10.4	7.0 ± 10.4	7.5 ± 9.3
Early vasopressor, n (%)	1 (2)	17 (100)	7 (30.4)	24 (60)	25 (27.8)
Vasopressor, n (%)	7 (14)	17 (100)	23 (100)	40 (100)	47 (52.2)
Sepsis, n (%)	10 (20)	0 (0)	20 (86.9)	20 (50)	30 (33.3)
Cultured organism in blood (P:A:M:O:N), n	3:4:1:1:1		13:3:0:1:3	13:3:0:1:3	16:7:1:2:4
Post-burn day of sepsis, days, mean ± SD	17.5 ± 13.9		13.0 ± 8.6	13.0 ± 8.6	14.5 ± 10.6
LOS, days, mean ± SD	66.0 ± 28.2	2.5 ± 1.1	19.8 ± 15.1	12.4 ± 14.3	42.2 ± 35.2

Early deaths, non-survivors who developed burn shock in spite of fluid resuscitation and died within 3 days of burn injury; Late deaths, mortality caused by other than burn shock; FB, flame burn; SB, scalding burn; EB, electrical burn; CB, chemical burn; SKB, spark burn; AKI: acute kidney injury; Early AKI, AKI that developed within 5 day of injury; BS, burn shcok; RD, rhabdomyolysis; SS, septic shock; MD, medication; TBSA, total body surface area: CRRT, continuous renal replacement therapy; RIFLE, risk, injury, failure, sustained loss, and end-stage kidney disease; RIFLE (R:I:F), patient RIFLE criteria for patients starting CRRT; Early vasopressor, vasopressor infusion due to shock within 1 day of admission; Cultured organism: organism cultured in blood from paitents with sepsis; P: *Pseudomonas aeruginosa*; A: *Acinetobacter baumanii*; M: multi-resistant *Staphylococcus aureus*; O: Other organisms; N: blood culture negative; LOS, length of hospital stay.

**Table 3:** Performance characteristics of biochemical markers with for predicting acute kidney injury (AKI)

**(a) Performance characteristics of biochemical markers for predicting early AKI development (n =66)**						
	**Time from admission**					
	**0 h**	**3 h**	**6 h**	**12 h**	**24 h**	**48 h**
AUC of serum creatinine	0.816*	0.823*	0.881*	0.988*	0.987*	0.898*
Cut-off value, mg/dL	0.85	0.85	0.85	1.05	1.15	0.95
Sensitivity	0.710	0.645	0.700	0.926	0.913	0.778
Specificity	0.706	0743	0.824	0.971	1.000	0.853
AUC of serum cystatin C	0.615	0.54	0.605	0.746*	0.731*	0.619
Cut-off value, mg/L				0.70	0.75	
Sensitivity				0.704	0.652	
Specificity				0.500	0.618	
AUC of plasma NGAL	0.832*	0.905*	0.926*	0.847*	0.818*	0.705*
Cut-off value, ng/mL	100.5	172.0	159.0	175.5	185.5	144.0
Sensitivity	0.806	0.806	0.900	0.815	0.739	0.667
Specificity	0.735	0.800	0.794	0.765	0.912	0.647
AUC of urine NGAL	0.921*	0.927*	0.896*	0.813*	0.914*	0.802*
Cut-off value, ng/mL	36.6	31.9	26.8	26.8	42.3	52.9
Sensitivity	0.871	0.871	0.800	0.815	0.870	0.722
Specificity	0.824	0.829	0.853	0.765	0.824	0.706
**(b) Performance characteristics of biochemical markers for predicting late AKI development (n = 59)**						
	**Time from admission**					
	**0 h**	**3 h**	**6 h**	**12 h**	**24 h**	**48 h**
AUC of serum creatinine	0.581	0.55	0.609	0.605	0.660*	0.625
Cut-off value, mg/dL					0.85	
Sensitivity					0.739	
Specificity					0.676	
AUC of serum cystatin C	0.488	0.437	0.545	0.491	0.513	0.444
AUC of plasma NGAL	0.682*	0.658*	0.670*	0.605	0.571	0.537
Cut-off value, ng/mL	102.5	100.0	125.0			
Sensitivity	0.640	0.583	0.625			
Specificity	0.735	0.629	0.676			
AUC of urine NGAL	0.692*	0.681*	0.644	0.599	0.598	0.604
Cut-off value, ng/mL	11.95	7.25	8.25			
Sensitivity	0.680	0.708	0.625			
Specificity	0.647	0.600	0.588			

Early AKI, development of AKI within 5 days of injury; late AKI, development of AKI beyond 5 days after injury; AUC, area under the receiver operating characteristic curve; NGAL, neutrophil gelatinase-associated lipocalin. *Statistically significant difference (*P* < 0.05).

**Table 4:** Performance characteristics of biochemical markers for predicting mortality

**(a) Performance characteristics of biochemical markers for predicting early death (n = 67)**						
	**Time from admission**					
	**0 h**	**3 h**	**6 h**	**12 h**	**24 h**	**48 h**
AUC of serum creatinine	0.699*	0.697*	0.747*	0.960*	0.941*	0.946*
Cut-off value, mg/dL	0.85	0.85	0.85	1.15	1.65	0.95
Sensitivity	0.588	0.529	0.625	0.923	0.889	1.000
Specificity	0.640	0.640	0.694	0.898	0.980	0.776
AUC of serum cystatin C	0.636	0.501	0.581	0.812*	0.757*	0.811*
Cut-off value(mg/L)				0.80	0.81	0.79
Sensitivity				0.692	0.667	0.750
Specificity				0.694	0.735	0.714
AUC of plasma NGAL	0.848*	0.919*	0.920*	0.892*	0.804*	0.776
Cut-off value, ng/mL	144.5	191.5	181.0	177.0	185.5	
Sensitivity	0.824	0.882	0.938	0.923	0.778	
Specificity	0.760	0.820	0.837	0.702	0.735	
AUC of urine NGAL	0.953*	0.984*	0.967*	0.940*	0.972*	0.939*
Cut-off value, ng/mL	98.9	172.0	45.5	46.0	50.0	83.8
Sensitivity	0.941	0.941	1.000	1.000	1.000	1.000
Specificity	0.840	0.920	0.878	0.796	0.878	0.796
**(b) Performance characteristics of biochemical markers for predicting late death (n = 73)**						
	**Time from admission**					
	**0 h**	**3 h**	**6 h**	**12 h**	**24 h**	**48 h**
AUC of serum creatinine	0.595	0.561	0.609	0.625	0.594	0.617
AUC of serum cystatin C	0.478	0.450	0.529	0.503	0.463	0.425
AUC of plasma NGAL	0.650*	0.765*	0.800*	0.629	0.534	0.515
Cut-off value. ng/mL	100.5	126.5	146.5			
Sensitivity	0.696	0.739	0.783			
Specificity	0.600	0.700	0.714			
AUC of urine NGAL	0.734*	0.691*	0.717*	0.607	0.635	0.583
Cut-off value, ng/mL	20.1	16.7	15.6			
Sensitivity	0.696	0.696	0.739			
Specificity	0.640	0.640	0.673			

Early death, non-survivors who developed burn shock despite fluid resuscitation and died within 3 days of burn injury; late death, mortality due to causes other than burn shock beyond 3 days; AUC, area under the receiver operating characteristic curve; NGAL, neutrophil gelatinase-associated lipocalin. *Statistically significant difference (*P* < 0.05).

**Table 5:** Multivariate binary logistic regression analysis for acute kidney injury (AKI) development and mortality at all time points within 48 h of admission

**(a) Multivariate logistic regression analysis of AKI development**				
			**95% CI for odds ratio**	
	**Odds ratio**	***P*-value**	**Lower**	**Upper**
Age >60 years			3.107	12.117
TBSA burned >50%	8.628	0.000*	5.036	14.783
Sex, female	4.199	0.000*	1.940	9.086
Inhalation injury present	3.129	0.000*	1.734	5.647
Serum creatinine >1.1 mg/dL	74.930	0.000*	7.926	708.331
Serum cystatin C >1.1 mg/L	0.150	0.038*	0.025	0.902
Plasma NGAL >153 ng/mL	1.924	0.016*	1.128	3.282
Urine NGAL >131 ng/mL	2.568	0.009*	1.263	5.220
**(b) Multivariate logistic regression analysis of mortality**				
			**95% CI for odds ratio**	
	**Odds ratio**	***P*-value**	**Lower**	**Upper**
Age >60 years	23.322	0.000*	9.885	55.023
TBSA burned >50%	25.821	0.000*	12.037	55.389
Sex, female	4.704	0.000*	1.999	11.070
Inhalation injury present	0.923	0.771	0.536	1.588
Serum creatinine >1.1 mg/dL	0.976	0.947	0.480	1.986
Serum cystatin C >1.1 mg/L	2.562	0.137	0.741	8.853
Plasma NGAL >153 ng/mL	1.886	0.023*	1.091	3.259
Urine NGAL >131 ng/mL	7.854	0.000*	3.949	15.622

The biomarker cut-off values were designated according to normal reference ranges, regardless of our results. The model was based on the univariate analysis, and no significant interactions between correlated variables were found in a correlation matrix. *Statistically significant difference (*P* < 0.05).

## Results

### Patient characteristics

The patients’ demographic and characteristic data, classified according to AKI development, are listed in Table 1. Of these patients, 35 did not develop AKI, 31 were classified into the early AKI group, and 24 were classified into the late AKI group. There were 22 patients diagnosed according to creatinine criteria alone, 2 were diagnosed according to urinary criteria alone, and 26 were diagnosed according to both creatinine and urinary criteria.

The sex and age were not significantly different among the groups. However, the mean % TBSA burned, presence of inhalation injury, mechanical ventilation, rhabdomyolysis, early vasopressor infusion, sepsis development, length of hospital stay, and mortality differed significantly between the groups. The early AKI group exhibited the most severe burn injuries, whereas the burn injuries were more severe in the late AKI group compared to the non-AKI group.

Table 2 shows the patients’ demographic and characteristic data classified according to mortality. There were 50 survivors and 40 non-survivors. Among the 40 non-survivors, 17 died from burn shock within 3 days of burn injury. Patients with early deaths exhibited the most severe burn injuries, with a mean % TBSA burned of 82.9% and a mean length of hospital stay of only 2.5 days.

### Changes in the levels of four biomarkers during 48 h after admission with respect to AKI development

The changes in the levels of each biomarker (serum creatinine, serum cystatin C, plasma NGAL, and urine NGAL) over time were compared between the groups (Figure 1). At all time points, the serum creatinine and plasma and urine NGAL levels were significantly higher in the early AKI group than in the non-AKI group. However, the cystatin C levels were significantly higher only at 12 and 24 h after injury when comparing the early AKI and non-AKI groups.

To estimate the predictive power of each biomarker, we performed a ROC curve analysis for early AKI (Table 3(a)). The significant cut-off values for serum creatinine for predicting early AKI ranged from 0.85 to 1.15 mg/dL between the time points of 0 and 48 h. The serum cystatin C levels were significant only at 12 and 24 h, and the cut-off values ranged from 0.7 to 0.75 mg/L. The plasma NGAL cut-off values for predicting early AKI ranged from 100.5 to 185.5 ng/mL and the urine NGAL cut-off values ranged from 26.8 to 52.9 ng/mL.

When we compared the non-AKI and late AKI groups with respect to each biomarker, the serum creatinine and cystatin C levels did not differ at any time point. However, the plasma and urine NGAL levels differed significantly between the non-AKI and late AKI groups only during the hyper-acute period (plasma NGAL: 0, 3, and 6 h after injury; urine NGAL: 0 and 3 h after injury).

In the ROC curve analysis for predicting late AKI, only the plasma and urine NGAL levels remained significant for up to 6 h from injury (Table 3(b)). The cut-off values for plasma NGAL ranged from 100.0 to 125.0 ng/mL and the cut-off values for urine NGAL ranged from 7.25 to 11.95 ng/mL.

### Changes in the levels of four biomarkers during 48 h after admission with respect to mortality

The levels of each biomarker (serum creatinine, serum cystatin C, plasma NGAL, and urine NGAL) were compared between the groups classified according to mortality. At nearly all the time points, the serum creatinine, plasma NGAL, and urine NGAL levels were significantly higher in the early-death group than in the survivors group (Figure 2). However, the cystatin C levels were significantly higher in the early-death group only after 12 h. In the ROC curve analysis for predicting early death, the cut-off values for serum creatinine ranged from 0.85 to 1.65 mg/dL, those for plasma NGAL ranged from 144.5 to 191.5 ng/mL and those for urine NGAL ranged from 45.5 to 172.0 ng/mL. The cut-off values for plasma NGAL ranged from 0.79 to 0.81 mg/L (Table 4(a)).

When we compared the late-death and survivor groups with respect to each biomarker, no differences were found in the serum creatinine and cystatin C levels at any time point. Only the plasma and urine NGAL levels differed significantly between the late death and survivor groups within 6 h of burn injury. In the ROC curve analysis for late death (Table 4(b)) the significant cut-off values for plasma NGAL ranged from 100.5 to 146.5 ng/mL within 6 h of admission. Additionally, the cut-off values for urine NGAL ranged from 15.6 to 20.1 ng/mL within 6 h of admission.

### Changes in plasma and urine NGAL levels with respect to burn size and inhalation injury

The presence of inhalation injuries and the % TBSA burned are the most powerful factors with which to determine the injury severity in major-burn patients. Therefore, we investigated the differences in the plasma and urine NGAL levels with respect to the injury severity as determined by the % TBSA burned and the presence of inhalation injuries. According to our data, as the % TBSA burned increased (larger burn-size group: 50 to 100% TBSA burned), both the mean plasma NGAL and urine NGAL levels significantly increased within 12 h of admission in both patients with and without inhalation injuries (Figure 3).

As shown in Figure 3, we observed gross difference in plasma and urine NGAL with inhalation injury. In the patients with TBSA burned between 20 and 49%, mean plasma NGAL was 116.1 in the patients without inhalation injury and 176.8 in those with inhalation injury (*P* = 0.018). Also, mean urine NGAL in these patients was significantly different with inhalation injury (49.4 without inhalation injury versus 172.4 with inhalation injury, *P* = 0.048). In the patients with TBSA burned between 50 and 100%, there were significant differences in plasma NGAL (211.1 without inhalation injury versus 267.5 with inhalation injury, *P* = 0.013) and urine NGAL (227.0 without inhalation injury versus 556.3 with inhalation injury, *P* = 0.000) by the presence of inhalation injury.

After classifying the patients into subgroups according to 50% TBSA burned, we investigated changes in the plasma and urine NGAL levels with respect to AKI and mortality. In both groups (< 50% and ≥ 50% TBSA burned), the plasma NGAL levels differed significantly with respect to AKI (no AKI versus AKI; < 50% burned, 103.0 versus 169.1 ng/mL, *P* < 0.001; ≥ 50% burned, 137.6 versus 260.0 ng/mL, *P* < 0.001). Similarly, the urine NGAL levels differed significantly with respect to AKI (< 50% burned, 32.1 versus 137.2 ng/mL, *P* <0.001; ≥ 50% burned, 114.0 versus 438.2 ng/mL, *P* < 0.001; Figure 4(a), 4(b)). In the same subgroups, the plasma NGAL levels differed significantly with respect to mortality (survivors versus non-survivors < 50% burned, 116.2 versus 172.9 ng/mL, *P* = 0.018; ≥ 50% burned, 117.3 versus 201.8 ng/mL, *P* < 0.001). Similarly, the urine NGAL levels differed significantly according to mortality (< 50% burned, 48.5 versus 178.5 ng/mL, *P* = 0.049; ≥ 50% burned, 59.9 versus 554.1 ng/mL, *P* < 0.001; Figure 4(c), (d)).

### Multivariate logistic regression analysis for predicting AKI and mortality

A multivariate binary logistic regression analysis for predicting AKI and mortality was performed and included the laboratory results at all time points. The age groups were categorized as either <60 or ≥60 years, the % TBSA burned groups as <50% or ≥50% of % TBSA burned, the serum creatinine levels as <1.1 or ≥1.1 mg/dL, the serum cystatin C levels as <1.1 or ≥1.1 mg/L, the plasma NGAL levels as <153 or ≥153 ng/mL, and the urine NGAL levels as <131 or ≥131 ng/mL. The cut-off values of the biomarkers were determined according to normal reference ranges from non-hospitalized donors. The analysis model was based on the univariate analysis, and no significant interactions were found between correlated variables in a correlation matrix.

All variables, including age, % TBSA burned, sex, inhalation injury, serum creatinine levels, serum cystatin C levels, plasma NGAL levels, and urine NGAL levels, were independently associated with AKI development (Table 5(a)). Age, sex, % TBSA burned, plasma NGAL levels, and urine NGAL levels were also independently associated with mortality. However, inhalation injury, serum creatinine levels, and serum cystatin C levels were not independently associated with mortality (Table 5(b)).

## Discussion

In this study, we evaluated the diagnostic utility of cystatin C, plasma NGAL, and urine NGAL levels in the early post-burn period for predicting AKI and mortality in patients with major burn injuries. In massively burned patients, the mechanisms associated with early AKI development and early death differ considerably from the mechanisms associated with late AKI development and late death. Therefore, this analysis evaluated both early and late AKI and both early and late deaths.All four markers (serum creatinine, serum cystatin C, plasma NGAL, and urine NGAL) were useful for predicting early AKI and early deaths at nearly all the investigated time points (Figures 1 and 2). However, the serum creatinine and cystatin C levels rapidly increased only after 12 h from admission. In contrast, the plasma and urine NGAL levels had already increased rapidly at the time of admission. During early AKI in massively burned patients, the urine NGAL levels first increased, followed by the plasma NGAL, cystatin C, and serum creatinine levels. Based on these results, we suspected that serum creatinine could not be used to diagnose the hyper-acute stage of AKI and that the plasma and urine NGAL levels were superior biochemical markers for diagnosing AKI. However, in patients with larger burn-wound surface areas, we observed increases in both the plasma and urine NGAL levels, as shown in Figure 3. This indicates that the % TBSA burned is a possible confounding factor for predictions of early AKI and early death. However, even in the subgroups classified according to the % TBSA burned values (<50% or ≥50%), the plasma and urine NGAL levels differed significantly with respect to AKI and mortality (Figure 4).

Only the plasma and urine NGAL levels exhibited statistical significance for predicting late AKI and late deaths within 6 h of admission. However, given these results, we cannot confirm that the plasma and urine NGAL levels can distinguish those patients who will develop late AKI and experience late death from those who will not develop AKI and will survive. The mean % TBSA burned values were 38.4% ± 14.1% in the no-AKI group versus 61.5% ± 19.2% in the late-AKI group and 41.9% ± 15.2% in the survivor group versus 65.7% ± 18.4% in the late-death group. All of the AUCs for the plasma and urine NGAL levels within 6 h were <0.7 for late AKI and late death. Therefore, we cannot confirm that the plasma and urine NGAL levels can predict late AKI development and late death in massively burned patients because of the possible confounding effect of the burn surface area. The cut-off values for urine NGAL were considerably lower than the normal reference range (Tables 3 and 4), suggesting that patients with intact renal function experienced significant diuresis due to a massive fluid resuscitation. Therefore, these cut-off values are not necessarily transferable to other situations.

In a multivariate logistic regression analysis that included the laboratory results at all time points, all the variables - including age, % TBSA burned, sex, presence of inhalation injury, and serum creatinine, serum cystatin C, plasma NGAL, and urine NGAL levels - were independently associated with AKI development (Table 5(a)). Age, sex, % TBSA burned, plasma NGAL levels, and urine NGAL levels were also independently associated with mortality. However, inhalation injury, serum creatinine levels, and serum cystatin C levels were not independently associated with mortality (Table 5(b)). Based on these results, we can confirm that the plasma and urine NGAL levels in the early post-burn period can predict AKI and mortality in major-burn patients. Our results are consistent with two recent reports demonstrating NGAL as an indicator for AKI in burn patients [14,15].

A limitation of this study is that early AKI was already present at admission, and therefore, the utility of refining the AKI diagnosis is unclear. The very good diagnostic performance of the creatinine levels at 12 to 24 h is unsurprising, as this test represents the gold standard for early AKI diagnosis. However, the poor gold standard for AKI assessment limits the performance characteristics of even the best biomarkers.

## Conclusions

Massively burned patients who maintain high plasma and urine NGAL levels until 48 h after admission are at risk of early AKI development and early mortality with burn shock. However, the plasma and urine NGAL levels in the early post-burn period failed to predict late AKI development and late death in this study. Nonetheless, the plasma and urine NGAL levels within 48 h of admission were independently associated with AKI development and mortality. In a future study, we should further investigate the diagnostic efficacy of plasma and urine NGAL levels in massively burned patients during the late post-burn period with regard to AKI and mortality.

## Key messages

• In massively burned patients, the plasma and urine NGAL levels increased rapidly during early AKI, followed by the cystatin C and serum creatinine levels.

• Increased plasma and urine NGAL levels in the early post-burn period were associated with early AKI and burn shock mortality but failed to predict late AKI and non-burn shock mortality.

• The plasma and urine NGAL levels increased significantly in patients with larger burn-wound surface areas.

• The plasma and urine NGAL levels within 48 h of admission were independently associated with AKI development and mortality.

## Abbreviations

AKI: acute kidney injury; AUC: area under the curve; BS: burn shock; CB: chemical burn; Cr: Creatinine; CRRT: continuous renal replacement therapy; EB: electrical burn; FB: flame burn; GFR: glomerular filtration rate; LOS: length of hospital stay; MD: medication; NGAL: neutrophil gelatinase-associated lipocalin; RD: rhabdomyolysis; RIFLE: risk injury, failure, sustained loss, and end-stage kidney disease; ROC: receiver operating characteristic; SB: scalding burn; SKB: spark burn; SS: septic shock; TBSA: total body surface area.

## Competing interests

The authors declare that they have no competing interests.

## Authors’ contributions

HTY and HSK developed the study concept and design. HY, YSC, DK, JH, JHK, and WC performed the acquisition of data and analysis and the interpretation of the data. HTY and HSK wrote the manuscript. All authors read and approved the final manuscript for publication.
